# High N availability decreases N uptake and yield under limited water availability in maize

**DOI:** 10.1038/s41598-023-40459-0

**Published:** 2023-08-31

**Authors:** Nora E. Flynn, Louise H. Comas, Catherine E. Stewart, Steven J. Fonte

**Affiliations:** 1 Department of Soil and Crop Sciences, Colorado State University, Fort Collins, CO 80523 USA; 2 Water Management and Systems Research Unit, USDA Agricultural Research Service, 2150 Centre Avenue, Bldg D Suite 320, Fort Collins, CO 80526 USA; 3 Graduate Degree Program in Ecology, Colorado State University, Fort Collins, CO 80523 USA; 4 Soil Management and Sugar Beet Research Unit, USDA Agricultural Research Service, Fort Collins, CO 80526 USA

**Keywords:** Plant sciences, Environmental sciences

## Abstract

Water and nitrogen (N) are the most limiting factors to plant productivity globally, but we lack a critical understanding of how water availability impacts N dynamics in agricultural systems. Plant N requirements are particularly uncertain when water is limited because of the interactive effect of water and N on plant growth, N demand, and plant uptake. We investigated impacts of N application and water availability on plant growth and N movement, including above and belowground growth, water productivity, N productivity, N uptake, N recovery, and greenhouse gas emissions within a semi-arid system in northeastern Colorado, USA. Moderately high soil N availability depressed grain yield and shoot growth under both limited and full water availability, despite no indication of physical toxicity, and came with additional risk of deleterious N losses. Under low N availability, plant N concentrations in aboveground tissues showed greater recovery of N than what was applied in the low N treatments under both full and limited water availability. This enhanced recovery underscores the need to better understand both plant soil foraging and processes governing resource availability under these conditions. Finally, limited water availability reduced N uptake across all N treatments and left 30% more soil nitrate (NO_3_^−^) deep in the soil profile at the end of the season than under full water availability. Our results show that plant N needs are not linearly related to water use and emphasize the need for an integrated understanding of water and N interactions, plant foraging for these resources, and the dynamics of processes that make N available to plants.

## Introduction

Decreasing agricultural water availability combined with increasing food demand suggests a critical need to improve agricultural water use efficiency^1^. There is a concurrent need to optimize N inputs due to the high fertilizer costs and multiple negative environmental impacts, including water quality degradation and greenhouse gas emissions^2^. Maize (*Zea mays*) is a globally important crop that is frequently limited by water and N supply. In eastern Colorado, maize is the dominant irrigated crop. However, groundwater depletion, variable snowpack, and increasing urban and industrial water demands are straining water supplies throughout the region^3–6^. Since water and N management challenges frequently co-occur, studies that consider these factors simultaneously offer great promise for improving resource use efficiency and reducing environmental impacts.

The timing of water availability affects plant water productivity (plant yield per unit of water used by the crop) because plant reproduction is often more sensitive to water limitations at specific growth stages, such as at pollination and seed set^7–10^. Conversely, maize grain yields are found to be less impacted when water limitations are experienced during vegetative stages, especially during late vegetative stage after plant establishment and initial growth is secure^7, 8^. However, water limitations and their timing also affect shoot and root system development, which in turn can affect plant N requirements.

Plant N requirements are uncertain when water is limited because of the interactive effects of water and N on plant growth, plant N demand, and plant uptake. For example, plants grown under limited water availability usually have smaller vegetative biomass, especially in proportion to the root system, because cell expansion in shoots is more sensitive to water limitations than that in roots^11^. This resulting shift in root:shoot allocation may conversely reduce plant demand for N while increasing foraging for soil resources. At the same time, the ability of roots to take up N is dependent on water availability^12^, so lower soil moisture could potentially hinder N uptake. Significant efforts have been made to optimize N management of water-limited maize^13–17^, and determine the reduction in N that can achieve maximum growth and yield under water limitation. However, determining the optimal reduction in N application for maize under water limitation is not straightforward, as some studies suggest that additional N applied during water limitation can increase maize grain yield and water use efficiency^14, 18–20^.

Water management plays a vital role in the environmental fate of N fertilizers by altering N movement and transformations in the soil^21–24^. Reduced soil moisture can decrease N_2_O emissions from soil^25^. However, if plants take up less N when water is limited, the amount of residual soil N increases, which could also lead to greater gaseous losses of N and NO_3_^−^ loss via leaching when soil moisture increases later in the season. Therefore, it is critical to understand not only how water availability impacts crop growth, but also the fate of N in agroecosystems since soil water availability has the potential to alter the form and size of N losses from the soil.

To improve our ability to predict optimal water and N additions, we established a field experiment where maize was grown under full and limited water availability at three levels of N availability (high, medium and low). The goals of this study were to: (1) test if N and water availability have interacting effects on maize yield, water and N productivity, and (2) understand the implications of water availability for N movement and loss from soils (above and belowground N uptake, N leaching, and N_2_O emissions) in a subsurface drip-irrigated maize system. We hypothesized that (1) limited water would increase water productivity but reduce vegetative biomass so that (2) N uptake would be reduced under limited compared to full water and that (3) less N uptake would result in increased residual soil N paired with greater potential for N loss.

## Methods

### Study site and experimental design

This experiment was conducted at the USDA-ARS Limited Irrigation Research Farm near Greeley, in Northern Colorado, USA (40° 26′ 57″ N, 104° 38′ 12″ W). The site is semi-arid with an elevation of 1427 m, and receives, on average, 215 mm of precipitation during the growing season (May–October) and 335 mm annually. Soils are predominately Olney fine sandy loam (fine-loamy, mixed, superactive, mesic Ustic Haplargids) with Otero sandy loam (coarse-loamy, mixed, superactive, calcareous, mesic Aridic Ustorthents) in small areas, with an average pH of 8.2. The field used for this study had maize the previous years under a uniform rate of subsurface drip irrigation and was strip-tilled annually.

In 2018, a field experiment with two water and three N fertilizer levels was established to investigate the combined impact of water and N availability on maize crop growth and N dynamics (Table 1; Supplementary Fig. S1). The limited water treatment achieved 75% of crop evapotranspiration (ET) during the late vegetative growth period from the vegetative stage with nine leaves per plant until tassel emergence (V9-VT) and 100% of ET for all other growth stages (Supplementary Fig. S2). This treatment was selected because it was previously shown to generate significant plant water savings without reducing yield^8^. The full water treatment received 100% of ET during the entire growing season. Target application rates for N treatments were based on recommendations for irrigated maize from pre-season soil sampling, which measured available soil N (46.2 kg ha^−1^) and 1.0% soil organic matter per soil mass in the surface layer (0–30 cm depth) prior to planting^26^. Based on this recommendation, a medium rate was set at 200 kg N ha^−1^, and low and high levels of N application were determined by subtracting or adding ~ 70 kg ha^−1^ from this recommended rate. All water and N treatment combinations were applied within a randomized split-plot design, with each treatment being present in four replicate blocks (Supplementary Fig. S1). Water treatments were applied to the main plots, and N treatments were applied to sub-plots within each water treatment plot. Sub-plots were 9 m wide and 20 m long, each containing 12 rows of maize (Syngenta hybrid N68B) planted at a density of 84,000 seeds ha^−1^, with 0.76 m spacing between rows.

**Table 1:** Precipitation, irrigation, evapotranspiration (ET), and N application data for two water treatments (full water, FW; limited water, LW) and three nitrogen treatment levels (high, H; medium, M; and low, L). Residual NO_3_-N in the top 30 cm of soil in April was 23.1 kg ha^−1^.

Water Trt	N Trt	Precipitation (mm)	Irrigation (mm)	Total water applied (mm)	ET (mm)	N from fertilizer (kg ha^−1^)	N from irrigation water (kg ha^−1^)	Total N applied (kg ha^−1^)
FW	H	104	497	601	581	132	149	281
	M	104	497	601	581	53	149	202
	L	104	497	601	581	14	149	163
LW	H	104	441	544	531	159	132	291
	M	104	441	544	531	81	132	213
	L	104	441	544	531	14	132	146

Before planting, the field was strip-tilled. An equal amount of starter fertilizer (16.8 kg N ha^−1^) was injected into rows with seeds using a combination of ammonium polyphosphate (10.1 kg N ha^−1^) and urea ammonium nitrate (UAN) (6.7 kg N ha^−1^). Subsequent N fertilizer was added by side-dressing UAN at the vegetative stage with four leaves present (V4, approximately four weeks after planting) to create N treatment differences (Table 1). Groundwater used for irrigation at the site contained 30 ppm NO_3_^−^, so additional side-dressed N fertilizer was reduced based on expected N supplied via irrigation. Flow-monitored sub-surface drip lines located approximately 23 cm below the soil surface were used to deliver irrigation throughout the season, based on target ET levels as determined by water balance using neutron probe and time domain reflectometry. An on-site weather station measured precipitation and allowed calculation of local ET.

### Plant and soil measurements

Plants were sampled in late September when plants reached reproductive maturity (physiological maturity, R6) to assess aboveground biomass and N uptake. This sampling time reflects the plant stage when all physiological activity (of importance here: N uptake and translocation) has essentially stopped, and plants begin to dry. The collection of plant material, complied with relevant institutional, national, and international guidelines and legislation. Five plants, located in a row with no skips or doubles, were cut at ground level in each plot, separated into stalks, leaves, and ears. Row lengths were measured to determine the ground area. Leaves were scanned with a leaf area meter (LI-3100C; LI-COR, Lincoln, Nebraska, USA). All plant components were oven-dried at 65 °C and weighed. Aboveground vegetative biomass included all aboveground parts (e.g., stalks, cobs, husks) except grain. Representative sub-samples of each oven-dried component were ground for subsequent elemental analyses. Grain yield was determined at plant maturity (R6) by collecting ears by hand from an area within the center of the plots, two rows wide and 5 m long. Grain was separated from the ears, weighed separately, and adjusted to 15.5% moisture content for standardization.

End-of-season soil cores (6.35 cm diameter) were collected in October 2018 to determine root biomass (kg ha^−1^) and available soil N at four depths (0–30, 30–60, 60–90, 90–120 cm). Four soil cores were collected with a Giddings probe in each plot: two in-row cores and two inter-row cores. Visible roots from each depth increment were removed by hand-picking from samples. Collected roots were then oven-dried, weighed, and sub-replicates averaged for each plot on a ground area basis. Row and between-row soil samples from each plot were composited within each depth, air-dried, and sieved to 2 mm. Stalks, leaves, grain, and root subsamples were analyzed for total C and N using a combustion analyzer (LECO Tru-SPEC, St. Joseph, MI). To determine soil NO_3_^−^ concentration at each depth, a 5 g of subsample homogenized field moist soil was extracted with 25 mL of 1 M KCl solution and measured with colorimetry^27^ with a UV–Vis Spectrophotometer (Shimadzu, Kyoto, Japan).

### Greenhouse gas measurements

Measurements of N_2_O emissions began in early May and ended in late September 2018. Two circular polyvinyl chloride anchors (20.3-cm-diameter) were placed in each plot (inserted to a depth of 10 cm) spanning half a row to capture spatial variability within rows. Lids with an airtight gasket affixed to the top of each anchor during each GHG sampling event. Samples were collected approximately twice a week during periods of frequent irrigation and less often after irrigation ended. Gas samples were collected in the morning between 8:00 h and 12:00 h to approximate average daily flux and minimize the effects of diurnal variation^28, 29^. During gas sampling, chambers were vented for atmospheric pressure and deployed for 45 min. Gas samples were collected starting at 0, 15, 30, and 45 min with 35 mL polypropylene syringes and immediately transferred to a 12 mL evacuated glass exetainer fitted with a screw cap and rubber butyl septum (Exetainer vial from Labco Limited, High Wycombe, Buckingham-shire, UK). Internal chamber temperature was measured using thermocouple wires installed in chamber lids with an airtight seal and used later to calculate gas abundance in the chamber. Samples were analyzed within two weeks of collecting with an automated gas chromatograph (Varian model 3800, Varian Inc., Palo Alto, CA) equipped with an electron capture detector.

### Analysis and calculations

Leaf area index (LAI) was calculated as the total leaf area per ground surface area based on the average of five plants sampled. Harvest index (HI) was calculated by dividing the oven-dry grain biomass by total aboveground plant oven-dry biomass (grain and vegetative biomass).

Evapotranspiration was calculated using weather station data and a water balance approach described in Trout and DeJonge^30^. Crop water productivity was calculated at grain yield divided by total water (ET) used by the crop. Nitrogen productivity was calculated as grain yield divided by total N applied (N in fertilizer and irrigation water). Total N uptake (kg ha^−1^) was calculated by multiplying the oven-dry biomass of each plant component (grain, shoots, and roots) by its corresponding N concentration and then summing all the aboveground parts. To examine total N recovery and account for the slightly different N application amount for each N level with the two irrigation treatments, we divided the total amount of N uptake by the total amount of N applied (in fertilizer and irrigation water).

### Greenhouse gas calculation

Because N_2_O emissions were low, we used linear regression to estimate gas fluxes to avoid overestimating fluxes^29, 31^. Cumulative fluxes for each soil chamber were calculated from the sum of measured and interpolated values of daily fluxes. Interpolated values for non-measured days were determined by linear interpolation using the following equation:

1 $${\text{Flux }} = {\text{ F1 }} + \, \frac{{({\text{F2}} - {\text{F1}})}}{{\left( {{\text{D2}} - {\text{D1}}} \right)}},$$

where F1 is the measured gas flux on the closest day before the day that requires interpolation, F2 is the measured gas flux on the closest day after the date of the computed flux. D1 is the day of the growing season on which F1 was measured, D2 is the day of the growing season on which F2 was measured.

The emissions factor was calculated to estimate the percent of N applied lost as N_2_O emissions. Typically, the emissions factor is calculated by subtracting the emissions of a control treatment from the treatment emissions. Because this experiment didn’t have a 0 N treatment, a modified emissions factor (EF) was calculated as:

2 $${\text{EF}} = \frac{{{\text{kg}}\,{\text{N}}_{{2}} {\text{O - N}}\,{\text{ha}}^{{ - {1}}} \,{\text{season}}^{{ - {1}}} }}{{{\text{kg}}\,{\text{N}}\,{\text{applied}}\,{\text{season}}^{{ - {1}}} }} \times { 1}00.$$

### Statistical analysis

Analysis was conducted on 21 plots of the original 24 plot experiment due to an irrigation line failure. This resulted in four replicates of each N level under limited water and three replicates of each N level under full water. We assessed the effect of irrigation, N, and the interaction of water and N availability using a two-way ANOVA. Water treatment was treated as a categorical variable, and N level was treated as a continuous variable. For yield and aboveground biomass responses, individual t-tests were run to test specific comparisons when the two-way ANOVA indicated significant overall differences. Root mass and residual NO_3_^−^ were assessed separately at each depth they were measured. Response variables were checked for normality and homogeneity of variance. Post hoc mean comparisons (Tukey–Kramer HSD test) were conducted to assess treatment differences when treatment effects were significant in the ANOVAs. Analyses were performed using R version 4.0.3. using the lme4, lmerTest, and emmeans packages^32, 33^.

## Results

### Water and N applications

Precipitation during the 2018 growing season was approximately half of the average growing season precipitation (Table 1), while temperatures were above average. Limited water treatment applied as 75% of full ET during the 4-week late vegetative period with full water requirements met during the remaining season resulted in the limited water treatment receiving 91% of full ET for the growing season (Table 1). Targeted levels of N were similar between water treatments (Table 1).

### Plant N uptake and N recovery

**Figure 1:**
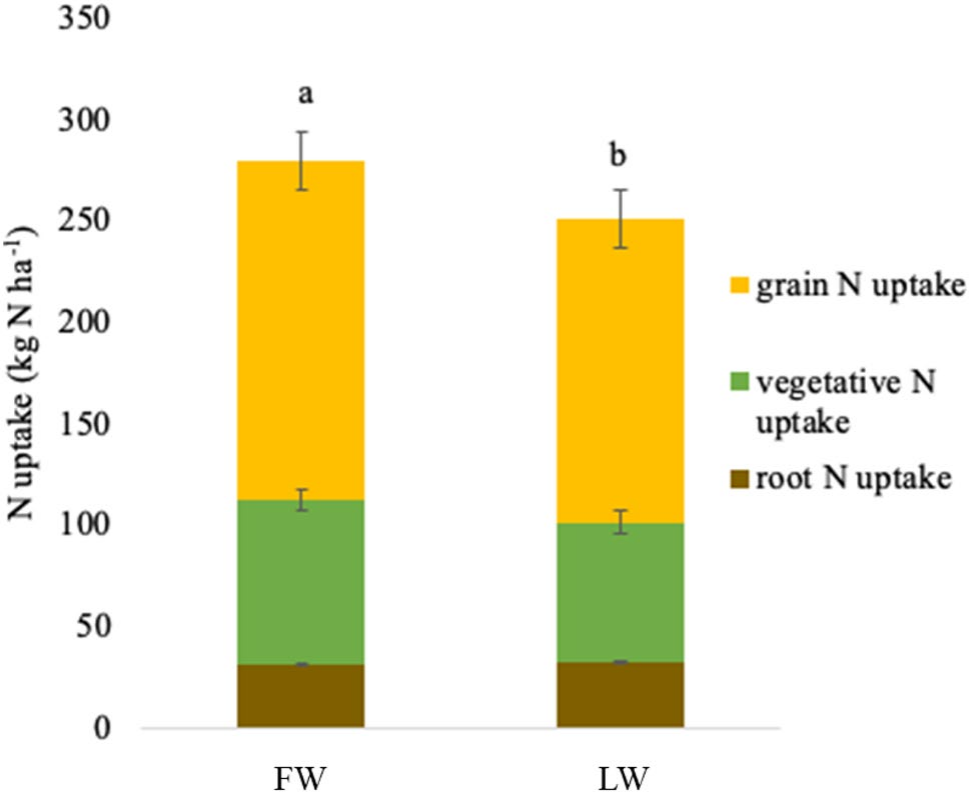
Mean N uptake in the roots, vegetative biomass, and grain under full water (FW) and limited water (LW). Different letters indicate a significant difference between FW and LW treatments in total recovery (the sum of root, vegetative, and grain N uptake, P < 0.05). Error bars represent standard error.

Limited water significantly reduced total maize N uptake by 10% compared to full water (Fig. 1; *P* < 0.05). The reduction in uptake was mainly driven by reduced biomass under limited water because when tissue concentrations differed among treatments, they were typically greater under limited compared to full water and higher when more N was applied (Table 2). Specifically, although leaf N concentrations were similar among irrigation and N treatments, stalk and root N concentrations were 17 and 20% higher, respectively, under limited compared to full water (*P* < 0.01 for both). Root and grain N concentrations were generally higher when more N was applied (*P* < 0.01and *P* = 0.02, respectively).

**Table 2:** Mean N concentration (%) of leaves and stems, roots and grain at physiological maturity (R6) for the same water and N levels described in Table 1. Values in parentheses show standard error. ANOVA P-values for each main effect are presented at the bottom of the table with significant effects (P < 0.05) in bold.

Water Trt	N Trt	Leaf N%	Stalk N%	Root N%	Grain N%
FW	H	1.43 (0.14)	0.61 (0.07)	1.35 (0.06)	1.26 (0.03)
	M	1.70 (0.04)	0.61 (0.05)	1.10 (0.08)	1.21 (0.02)
	L	1.63 (0.05)	0.65 (0.01)	1.24 (0.08)	1.19 (0.02)
LW	H	1.53 (0.08)	0.76 (0.02)	1.69 (0.06)	1.23 (0.01)
	M	1.53 (0.10)	0.80 (0.04)	1.53 (0.12)	1.24 (0.01)
	L	1.47 (0.06)	0.71 (0.04)	1.35 (0.09)	1.21 (0.01)
ANOVA					
Water		0.28	**< 0.01**	**< 0.01**	0.60
N		0.53	0.37	**< 0.01**	**0.02**
W × N		0.07	0.96	0.45	0.08

Limited water reduced the overall proportion of applied N recovered in the maize plants across N treatments (Table 3; *P* = 0.04). Plant N recovery was 7% higher under full than limited water and was 1.5–2 times higher in the low N relative to the high N treatment under both full and limited water. We note that the low N treatments under both full and limited water recovered substantially more N than even the total N that was applied to those treatments, indicating an alternative N source to the plants increased under low N.

**Table 3:** Mean N recovery ratio (N accumulation in plant material per total N from applied N), leaf area index (LAI), water productivity (WP; grain yield corrected to 15.5% moisture content per total crop ET), N productivity (NP; grain yield corrected to 15.5% moisture content per total N applied), cumulative N_2_O emissions (total across the growing season), and emissions factor (EF; %) for the same water and N treatments described in Table 1. Values in parentheses show standard error. Different letters indicate significant differences between N treatments within water treatments. ANOVA P-values for each main effect are presented at the bottom of the table with significant effects (P < 0.05) in bold. An observed—expected yield assessment of 0 indicates that the full expected yield was achieved (that the observed yield was the same as the expected yield that was estimated from total N that was applied and present as residual).

Water Trt	N Trt	N recovery ratio	LAI	WP (kg grain mm^−1^ ET)	NP (kg grain kg^−1^ N applied)	Cumulative N_2_O emission (g N_2_O-N ha^−1^)	EF (%)
FW	H	1.01 (0.07) a	4.7 (0.3)	23.1 (1.4)	47 (3) a	477 (76)	0.17 (0.03)
	M	1.47 (0.08) b	4.8 (0.1)	25.5 (0.6)	73 (2) b	629 (152)	0.31 (0.08)
	L	1.61 (0.10) c	4.6 (0.1)	22.9 (1.2)	81 (4) c	400 (40)	0.25 (0.020
LW	H	0.81 (0.04) a	3.9 (0.1)	21.2 (0.9)	38 (2) a	525 (71)	0.18 (0.02)
	M	1.18 (0.03) b	4.3 (0.2)	23.4 (1.0)	58 (3) b	373 (75)	0.17 (0.04)
	L	1.80 (0.04) c	4.3 (0.2)	24.0 (1.4)	87 (5) c	392 (59)	0.27 (0.04)
ANOVA							
Water		**0.04**	**< 0.01**	0.48	0.08	0.33	0.34
N		**< 0.01**	0.21	0.69	**< 0.01**	0.28	**0.05**
W × N		**0.03**	0.30	0.53	0.44	0.63	0.74

### Effects on grain yield, aboveground growth, and water and N productivity

**Figure 2:**
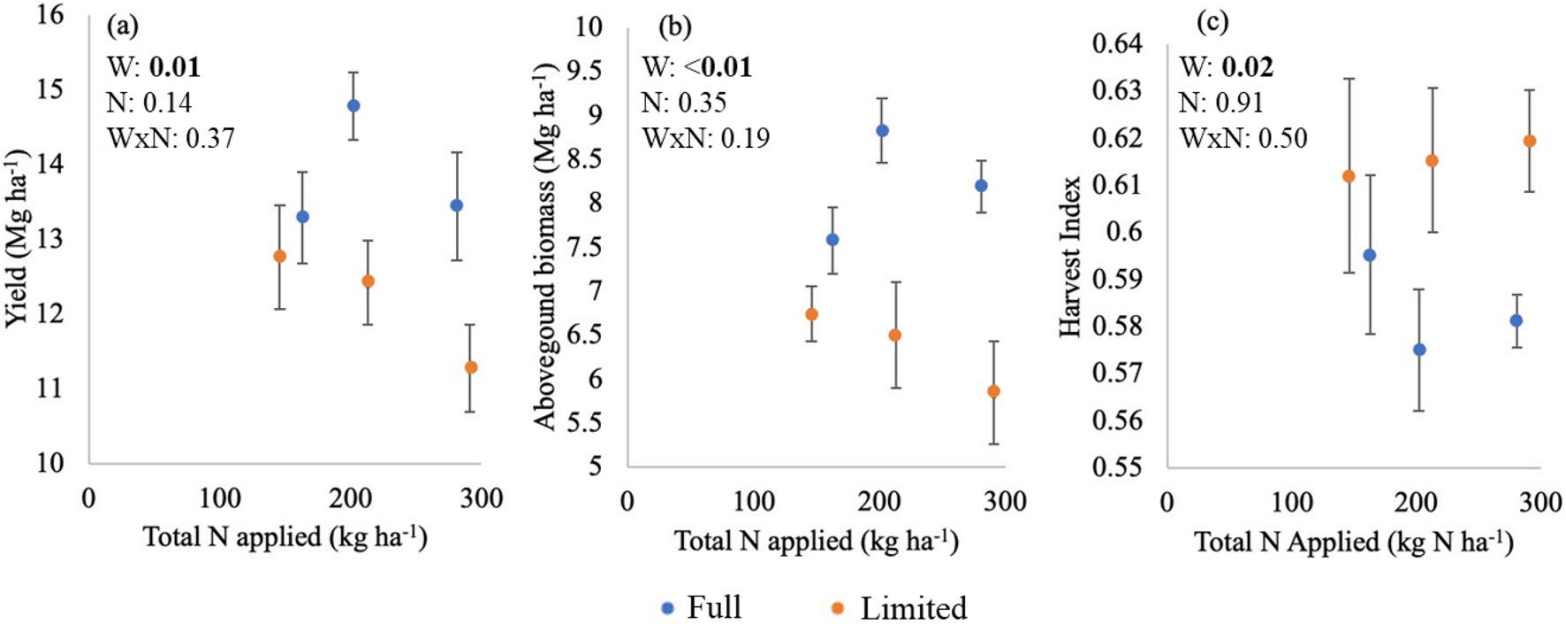
Average (**a**) yield, (**b**) aboveground biomass, and (**c**) harvest index (grain biomass divided by total aboveground biomass) under two water (full and limited) and three N nitrogen treatment levels. Points represent averages of individual water and N treatments, while error bars represent standard error among the replicates of each treatment. ANOVA P-values provide statistics on the main factors (water and N rate) as well as their interaction (Water × N) in the top left corner of each plot.

Grain yield decreased by 9% from medium to the highest N level across both irrigation treatments (Fig. 2a). High N treatment particularly depressed grain yield under limited water (individual t tests; full water/high N vs limited water/high N: P = 0.03; and limited water/low N vs limited water/high N: P = 0.05). Overall, limited water reduced grain yield by 12% compared to full water across all N treatments (Fig. 2a; *P* < 0.05). Limited water reduced aboveground biomass by 22% compared to full water (*P* < 0.01), with aboveground biomass following a similar tendance for reduced aboveground biomass from medium to the highest N level in both irrigation treatments (Fig. 2b). Like the reduction of aboveground biomass, LAI was also lower in response to limited water, but LAI of all treatments indicated full light interception (Table 3; Comas et al.^8^). With the biomass reduction in response to limited water being greater than the reduction in yield, HI increased by 5% under limited compared to full water (*P* = 0.02), with no significant N level effect on HI (Fig. 2c).

There was no difference in water productivity in response to water or N treatments (Table 3). However, the overall effect of limited water across N treatments was a 9% reduction in nitrogen productivity (Table 3), although the effect was only marginally significant (P = 0.08). Nitrogen productivity was 1.5–2 times lower for the high N than for the low N treatment under both full and limited water (*P* < 0.01).

### N movement and root growth

**Figure 3:**
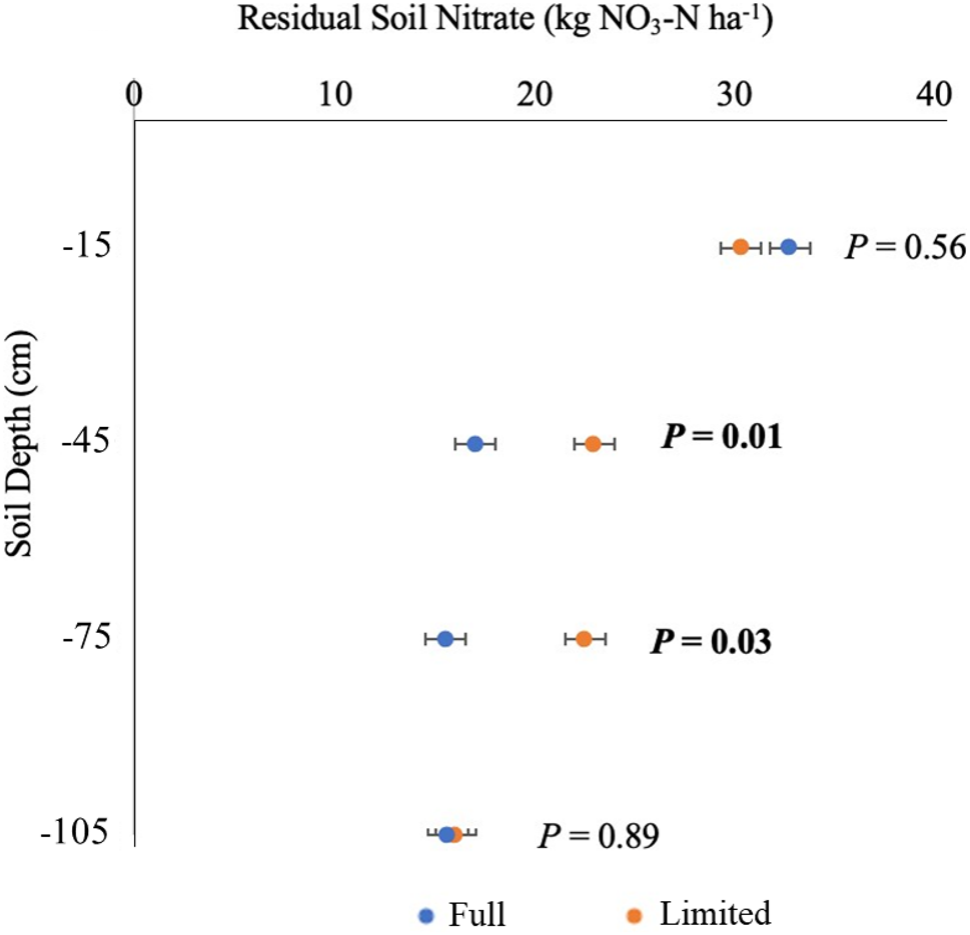
End-of-season residual soil NO_3_^−^ measured in terms of NO_3_-N (kg ha^−1^) at four depths (0–30, 31–60, 61–90, 91–120 cm) in a maize field averaged across N levels for the two water treatment levels (full and limited). Error bars represent standard error. ANOVA P-values for each depth are presented and bold represents a significant effect (P < 0.05).

Residual soil NO_3_^−^ at the end of the season was in highest concentration at the 0–30 cm depth in soil but with no differences among N treatments (Fig. 3). There was an overall effect of greater residual N in limited water treatments from 30 to 90 cm soil depth, where residual soil NO_3_^−^ increased by 22–35% in the limited compared to full water (Fig. 3). Residual soil NO_3_^−^ did not otherwise differ among soil depths and treatments.

Across all treatments, approximately 50% of root biomass was in the top 0–15 cm of the soil, 20% in the 15–30 cm, 20% in the 30–60 cm, and 10% in 60–90 cm (Table 4), with no roots below 90 cm. Water and N treatments did not influence root biomass except for at the 60–90 cm soil depth, where there was a significant interaction between water and N treatments such that under full water, root biomass decreased when less N was applied, but increased when less N was applied under limited water (Table 3). The greater root biomass under limited water and low N aligned with the 60–90 cm soil depth where residual N was also greater under limited water (Fig. 3).

**Table 4:** Mean standing root biomass (kg ha^−1^) by soil depth and total down to 90 cm depth for the same water and N treatments described in Table 1. Values in parentheses show standard error. Different letters indicate significant differences between N treatments. ANOVA *P*-values for each main effect are presented at the bottom of the table with significant effects (*P* < 0.05) in bold.

Water Trt	N Trt	Root biomass (kg ha^−1^) by soil depth				Total root biomass (kg ha^−1^)
		0–15 cm	15–30 cm	30–60 cm	60–90 cm	
FW	H	1251 (320)	603 (129)	575 (121)	393 (95) a	2824 (525)
	M	1552 (86)	351 (59)	614 (100)	160 (43) ab	2678 (35)
	L	1160 (398)	449 (126)	391 (85)	126 (44) b	2125 (565)
LW	H	797 (148)	555 (39)	573 (138)	73 (34) a	1998 (302)
	M	866 (305)	470 (11)	409 (43)	181 (24) b	1927 (354)
	L	1460 (411)	440 (81)	488 (36)	289 (54) b	2679 (419)
ANOVA						
Water		0.27	0.75	0.65	0.27	0.30
N		0.20	0.08	0.25	0.62	0.67
W × N		0.36	0.54	0.67	**< 0.01**	0.13

### N_2_O emissions

**Figure 4:**
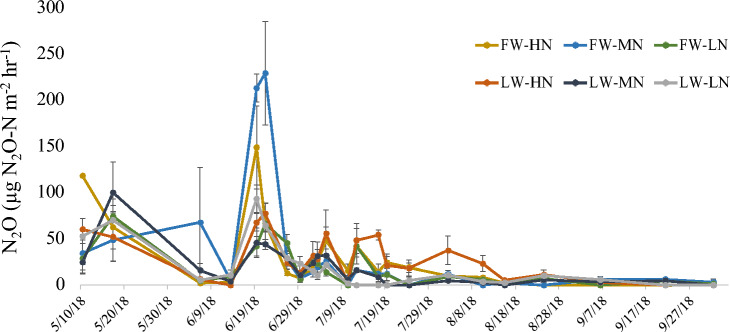
N_2_O emissions (µg N_2_O-N m^−2^ h^−1^) during the 2018 maize growing season for two water treatments (full water, FW; limited water, LW) and three nitrogen treatment levels (high, H; medium, M; and low, L). Limited water treatment occurred during the late vegetative growth stages (V9-VT), which occurred from July 3–31.

There were no significant treatment effects on cumulative N_2_O emissions (Table 3), although the highest peaks in N_2_O emissions occurred under full water with high and medium N application rates (Fig. 4). The timing of N emissions was generally similar among treatments with spikes in emissions following N application in mid-June. The highest N application rate under limited water continued to have small spikes through the July and part of August (Fig. 4).

As a result of little to no differences in cumulative N_2_O emissions among treatments, lower rates of N application resulted in proportionally greater emission losses relative to the amount of applied N (i.e., greater emissions factor, N_2_O emissions per unit N applied; Table 2).

### Water production functions and interactions with N

**Figure 5:**
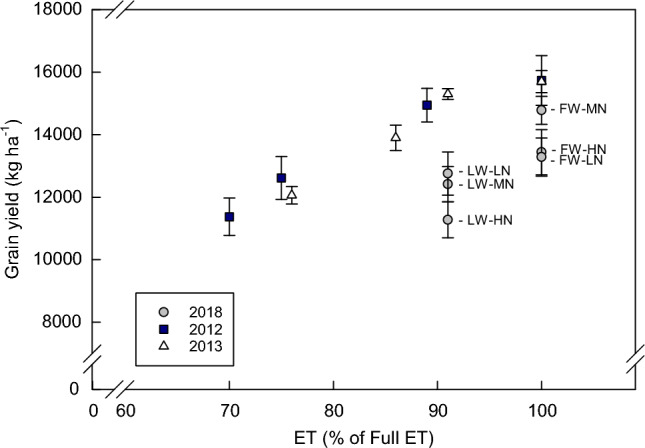
Water product functions (grain yield (moisture content) produced versus the ET used by the plants) of the treatments in this study (2018) and those from a previous study (2012 and 2013) conducted at the same site^8^. The ET of each treatment is presented as a percentage of that of the total ET used by the fully watered treatment of each year. Treatment labels from the current study are given in Table 1. The four irrigation treatments shown for 2012 and 2013 included a full water (FW) treatment and three limited water treatments that were targeted to supply 80, 65, and 50% of full ET in the late vegetative and grain-filling stages (see Comas et al.^8^ for full description).

There was nearly proportional reduction of yield in response to the reduction in crop water use (88% grain yield with overall 91% of the ETin limited *vs* full water; Fig. 5). High N was associated with yield reduction at both water levels but low N was also associated with a yield reduction at full water (Fig. 5).

## Discussion

Plant N requirements are particularly uncertain when water is limited because of the interactive effect of water and N on plant growth, plant N demand, and plant N uptake. Our data suggest that less N was needed to optimize yield under limited compared to full water. Interestingly, however, although low N application at full water was tied to yield reduction, higher N application was also tied to yield reductions at both full and limited water levels. These yield reductions are particularly relevant because studies have suggested that additional N applied during water limitation can increase maize grain yield and water use efficiency. Importantly, yield reductions in both full and limited water were found at moderate levels of NO_3_^−^ that plants typically tolerate and without any signs of physical toxicity^34^.

Counter to initial hypotheses, limited water strategically applied during the vegetative period did not increase water productivity, with potentially multiple explanations. Limited water availability reduced N recovery as hypothesized at medium and high N levels and led to greater soil residual, but, interestingly, N recovery at low N increased under limited compared to full water (Table 3). N recovery was not only greatest at low N application levels but exceeded the amount of N estimated to be available, suggesting that plants were either able to forage for residual pools of N in the soil profile or N mineralization was stimulated under low N application. Residual N at the end of the season was generally greater deep in the soil profile with limited water (Fig. 3), as hypothesized, but soil N_2_O emissions were generally low and similar across water and N treatments. Residual soil NO_3_^−^ left at the end of the growing season deep in the soil profile had increased potential for loss via leaching to groundwater, highlighting the need to precisely determine N needs under different irrigation management.

### Nitrogen uptake and recovery ratio

Maize grown under water-limited conditions typically requires less N to achieve maximum grain yield compared to well-watered maize^35^, likely because smaller plants need less N. The reduction in plant N uptake under limited water in this study was due to both to reduction in vegetative biomass as well as grain yield. Like the overall reduced N uptake, N recovery ratio at medium and high N application rates was lower under limited compared to full water, suggesting greater potential for N loss under limited water. Our ability to track the movement of specific sources on N is limited because our N recovery metric did not distinguish between fertilizer N versus N taken up from other soil sources (e.g., mineralized from soil organic matter). Conducting a similar experiment with isotope tracing of the various N sources in plants would allow for a more precise understanding of how limited water impacts the transformation and fate of soil N. However, the soil in this study was low in soil organic matter (SOM was 1% of soil mass in the top 30 cm), potentially limiting the total contribution of N via mineralization. It may be more likely that increased root growth deep in the profile under limited water and low N increased the plants ability to forage for N available deep in the soil profile.

While plant N uptake is largely determined by plant growth and N demand, soil N availability and root uptake capacity also play a critical role^36^. Nitrogen uptake depends on water availability with soil N moving towards the root surface by mass flow^12^, but under similar soil moisture, increasing the concentration of N in the soil should theoretically enhance N uptake. Increasing soil N concentration to increase N uptake, however, was not supported in this study, where increasing N application under limited water by 145 kg N ha^−1^ (from the low to high treatment) reduced N uptake. Under full water, increasing N by 118 kg ha^−1^ (from the low to high N treatment) also only resulted in an additional 22 kg ha^−1^ of N uptake by the plant, potentially because the additional N was also aligned with a reduction in plant biomass and grain yield (Fig. 2).

Limited water and nutrient availability encourages plants to invest in root biomass. Here, maize root biomass was greatest under low N and limited water at the deepest soil depth examined. Previous work at this field site has also shown limited water to increase maize root growth at depth^37, 38^. Interestingly, root biomass at the deepest depth was reduced limited water and high N, potentially due to the control of N on root system size (Table 4). The lack of an overall increase in root biomass under limited water in this study was in contrast to the findings in Flynn et al.^38^, which examined the impact of limited water on emissions at the same site in previous years, and potentially due to the fact that the deficit imposed in this study (75% of full ET) was milder than in the previous experiment (40% of full ET).

Most interestingly, however, plants in the low N treatments, under both full and limited water, recovered substantially more N than the total N that was applied to those treatments. Again, it is difficult to speculate on the source of this N without additional measurements. There is mounting evidence that crop plants get less than 50% of their N from the N fertilizer applied^39, 40^. It is unknown, though, how recent fertilizer application versus N mineralization versus residual N across the rooting zone (from past fertilizer application and mineralization) contribute to the total N uptake of crops and how this varies under different levels of N availability and fertilizer application. This information is critical for developing sustainable strategies for N management.

### Impact of N application and limited water on yield, water, and N productivity

Nitrogen is needed in large amounts by maize plants, so when N fertilizer prices are low or commodity prices are expected to be high, some farmers apply N fertilizers in excess as a form of insurance. Luxury N uptake has been shown to improve leaf longevity, lead to greater biomass, and reduce water stress impacts during maize grain filling^41^. However, excessively high or low N can have a negative effect on maize under limited water availability^42^. This curvilinear response (here, of both grain yield and aboveground plant biomass) to N application suggests hormesis^43^, where initially beneficial increases in N availability became negative after a point, despite N availability being at nontoxic levels, because N availability elicits plant responses that paradoxically may no longer aid plants in productivity. Several potential mechanisms could underlie a hormetic effect of N on plants. For example, too much N in maize leaves has been shown to close stomata, decrease stomatal conductance and, thus, negatively impact photosynthetic parameters and increase plant sensitivity to drought stress^42^. Excessive N rates have also been shown to reduce root growth and function and negatively impact crop growth^44, 45^.

In contrast to the results here, other studies have found increased N fertilizer application to benefit maize yield under water stress^14, 46^. In one of these studies in Egypt^46^, the highest N level applied to field grown maize was similar to the lowest N level in this study, such that all of the levels of N applied was less than what was needed to meet full plant N demand. In another study in the Great Plains of Colorado^14^, the optimal N level was found to be higher than here, but much greater planting density was used, such that a similar relative N requirement was found at full water. Moderately higher N increased water use efficiency under limited water in one of 3 years but excessive N also decreased water use efficiency. Here, under water limitation, grain production as well as N productivity, assessed as grain produced per N applied, was improved with lower N rates. Nitrogen fertilizer applied above 150 kg ha^−1^ under limited and 210 kg ha^−1^ under full water treatments appeared to have no additional benefit for yield. Thus, our results do not support the suggestion that high N increases WUE or otherwise benefits maize yield under water stress. However, the potential for N mineralization and amount of residual pools of N through the soil profile are critical to determining the optimal N fertilization.

Furthermore, applying limited water during the late vegetative growth stage reduced maize yield compared to full water and did not significantly improve water productivity water productivity. This is contrary to our hypothesis and previous studies that have demonstrated increased water productivity with strategic regulated limited water of maize^8–10^. Improving maize water productivity with limited water is challenging because maize is susceptible to water stress, and maize yield is often linearly related to ET^20, 47^. Using limited water only during the late vegetative stage, as we did in this study, is thought to be the ideal stage to reduce water use since yield is less sensitive to water stress at this stage than during early vegetative or anthesis and grain-filling^7, 16^. Limitations in water availability during the late vegetative stage can create a curvilinear water response function and increased water productivity (Comas et al.^8^; ~ 14% total savings in ET by applying approximately 71% of full ET in each of the late vegetative and maturation growth stages with no significant yield loss) (see data from this previous study in comparison the current study; Fig. 5). Interestingly, the yields in this study were lower and appeared to be more severely impacted by limited water than in previous studies at this location^8^. A different hybrid was used than in the previous study of limited water at this site. It is possible that this hybrid was more sensitive to limited water but these studies were conducted in different years and yields also vary among years. It is also possible that the water limitations imposed during the late vegetative stage of growth were extended too long, leading to negative impacts on yield because 50% of the plants were tasseling before the plants were taken out of water stress. Water stress is known to reduce yield in maize if it occurs during anthesis and early seed set^7^, and this water limitation may have impacted the 50% of the plants that were tasseling before water limitations were removed.

Water availability during the vegetative stage is a crucial determinant of leaf area and photosynthetic capacity, which strongly influences yield potential^10^. Leaf area in our study was reduced under limited compared to full water (Table 3), similar to Comas et al.^8^. However, plants under limited water were still capable of full light interception and, thus, should have had fully capacity for carbon fixation. Interestingly, here, HI, a measure of how efficiently C synthesized by the crop is allocated to grain versus vegetative biomass, was higher under limited than full water. This more efficient use of synthesized C represents a potentially desirable drought-tolerant trait, but overall, grain yield was still compromised by limited water. While our results do not support the idea that limited water improves water productivity, we agree with other studies that suggest more research is needed to discern how and when limited water can result in improved water productivity for maize^47, 48^.

### Residual nitrogen and N_2_O emissions

In line with the lower plant N uptake under limited water, we observed a corresponding buildup of NO_3_^−^ in the deeper soil layers (30–60 and 60–90 cm) under limited relative to full water after 1 year of treatment implementation. Others have also found end-of-season soil NO_3_^−^ to be higher under limited than full water in maize-based systems^16, 49, 50^. Drip irrigated systems, as used in this research, typically have lower fertilizer N leaching losses because water and nutrients are delivered directly to the rooting zone, resulting in overall lower deep percolation and increased synchrony of water application with plant demand^25^. In contrast, flood irrigated systems often have excess water being applied, especially at the top of the field, and thus more significant fertilizer leaching losses below the root zone^51, 52^.

Nitrogen remaining in the soil at the end of the season is vulnerable to multiple loss pathways, including via leaching and gaseous fluxes^21^, or can be immobilized and rereleased via mineralization for uptake by plants in subsequent seasons. While we did not follow the fate of the residual NO_3_^−^ after the growing season, we know that the amount of N leached from soil tends to increase with the amount of excess N applied^53^. Residual N at the end of the growing season is vulnerable to leaching, even in semi-arid environments, because even small amounts of water (e.g., less than 3 cm) can move soil NO_3_^−^ down 15–20 cm in a loamy sand soil^54^ and large spring rains frequently occur in the Central Plains region^55^. The end-of-season residual NO_3_^−^, therefore, represents a concern for environmental degradation but also an inefficient use of an expensive input that could impact the farmer’s net profits.

Water and N availability had no significant impact on cumulative N_2_O emissions in this study. This was surprising, as reduced water availability and soil moisture under limited water can decrease N_2_O emissions^25^ because both nitrification and denitrification are influenced by soil moisture^56^. Peak emissions do appear initially higher in the high and medium N rates of the full water treatment (Fig. 4). Multiple smaller peak in the high N rate of the limited water treatment later in the season contribute substantially to the cumulative emission of this treatment. One potential explanation is that the more extreme water limitation used in Flynn et al.^25^ (40% ET during the late vegetative stage) compared to the limited water treatment used here (75% ET during the late vegetative state) may be needed to reduce GHG emissions. At the same time, reducing N fertilizer inputs should reduce N substrates available for soil N_2_O producing processes, thus lowering N_2_O emissions^57, 58^. The lack of significant water and N treatment impacts suggests that our lowest irrigation and N levels did not meaningfully limit N_2_O producing processes, or alternatively, if low N fertilization promoted greater N mineralization, emissions contribution from N mineralization may have balanced the reduction in emissions from fertilizer sources.

It is also important to note that we only measured in-season N_2_O emissions. It is possible that in-season peaks were underestimated, but sampling occurred twice a week during weeks of active irrigation to minimize underestimations. However, it is possible off-season that accumulated residual soil N under limited water could lead to greater emissions compared to full water. Other factors controlling N_2_O emissions could also have suppressed our treatment effect, including compaction, temperature, pH, organic matter, and texture^59, 60^. Soil organic matter, which was 1% soil mass in the top 30 cm at this field site, may have been especially limiting. Soil carbon availability can limit N_2_O emissions because carbon is a key substrate and source of energy for microbial activity and associated soil N transformations^61^^.^

Despite having no treatment differences, the emissions factor were notably low in this study compared to other irrigated maize systems utilizing sprinkler irrigation because drip-irrigated systems are efficient at mitigating GHG emissions^62–65^. This resulted in an emissions factor much lower than the IPCC empirical estimate of 1%, which assumes a linear relationship between N applications and N_2_O emissions.

## Conclusion

Our findings illustrate the impact of water availability on N dynamics. We found no evidence that a high rate of N availability benefited plant growth under limited water availability. On the contrary, a high rate of N availability led to substantial decreases in grain yield under both limited and full water and came with additional risk of deleterious N losses. Clearly, N availability should be reduced when less water is available for crop production, but plant N requirements were not linearly related to water availability. In summary, our results emphasize the need for better understanding the complex dynamics of plant response to N and water availability, and N processes in soil that make N available to plants to advance sustainable N and water management.

### Supplementary Information

Supplementary Figures.

## Data Availability

The data that support the findings of this study are available from the corresponding author.

## References

[1] Wallace JS (2000). Increasing agricultural water use efficiency to meet future food production. Agric. Ecosyst. Environ..

[2] Robertson GP, Vitousek PM (2009). Nitrogen in agriculture: Balancing the cost of an essential resource. Annu. Rev. Environ. Resour..

[3] Kukal MS, Irmak S (2018). Climate-driven crop yield and yield variability and climate change impacts on the U.S. great plains agricultural production. Sci. Rep..

[4] Scanlon BR (2012). Groundwater depletion and sustainability of irrigation in the US High Plains and Central Valley. Proc. Natl. Acad. Sci. U. S. A..

[5] Warziniack T, Brown TC (2019). The importance of municipal and agricultural demands in future water shortages in the United States. Environ. Res. Lett..

[6] Wienhold BJ, Vigil MF, Hendrickson JR, Derner JD (2018). Vulnerability of crops and croplands in the US Northern Plains to predicted climate change. Clim. Change..

[7] Çakir R (2004). Effect of water stress at different development stages on vegetative and reproductive growth of corn. Field Crops Res..

[8] Comas LH, Trout TJ, DeJonge KC, Zhang H, Gleason SM (2019). Water productivity under strategic growth stage-based deficit irrigation in maize. Agric. Water Manag..

[9] Fereres E, Soriano MA (2007). Deficit irrigation for reducing agricultural water use. J. Exp. Bot..

[10] Geerts S, Raes D (2009). Deficit irrigation as an on-farm strategy to maximize crop water productivity in dry areas. Agric. Water. Manag..

[11] Hsiao TC, Xu LK (2000). Sensitivity of growth of roots *versus* leaves to water stress: biophysical analysis and relation to water transport. J. Exp. Bot..

[12] Ercoli L, Lulli L, Mariotti M, Masoni A, Arduini I (2008). Post-anthesis dry matter and nitrogen dynamics in durum wheat as affected by nitrogen supply and soil water availability. Eur. J. Agron..

[13] Ashraf U (2016). Maize growth, yield formation and water-nitrogen usage in response to varied irrigation and nitrogen supply under semi-arid climate. Turk. J. Field Crops..

[14] Eissa MA, Roshdy NMK (2019). Effect of nitrogen rates on drip irrigated maize grown under deficit irrigation. J. Plant Nutr..

[15] Hammad HM (2017). Maize plant nitrogen uptake dynamics at limited irrigation water and nitrogen. Environ. Sci. Pollut. Res..

[16] Kirda C (2005). Grain yield response and N-fertilizer recovery of maize under deficit irrigation. Field Crops Res..

[17] Mansouri-Far C, Ali S, Modarres M, Saberali SF (2010). Maize yield response to deficit irrigation during low-sensitive growth stages and nitrogen rate under semi-arid climatic conditions. Agric. Water Manag..

[18] Di Paolo E, Rinaldi M (2008). Yield response of corn to irrigation and nitrogen fertilization in a Mediterranean environment. Field Crops Res..

[19] Li Y (2019). A global synthesis of the effect of water and nitrogen input on maize (*Zea mays*) yield, water productivity and nitrogen use efficiency. Agric. For. Meterol..

[20] Pandey RK, Prof JWM, Admou A (2000). Deficit irrigation and nitrogen effects on maize in a Sahelian environment I. Grain yield and yield components. Agric. Water Manag..

[21] Barakat M, Cheviron B, Angulo-Jaramillo R (2016). Influence of the irrigation technique and strategies on the nitrogen cycle and budget: A review. Agric. Water Manag..

[22] Chilundo M, Joel A, Wesström I, Brito R, Messing I (2016). Effects of reduced irrigation dose and slow release fertilizer on nitrogen use efficiency and crop yield in a semi-arid loamy sand. Agric. Water Manag..

[23] Rimski-Korsakov H, Rubio G, Lavado RS (2009). Effect of water stress in maize crop production and nitrogen fertilizer fate. J. Plant Nutr..

[24] Sanchez-Martin L, Vallejo A, Dick J, Skiba UM (2008). The influence of soluble carbon and fertilizer nitrogen on nitric oxide and nitrous oxide emissions from two contrasting agricultural soils. Soil Biol. Biochem..

[25] Flynn N (2022). Deficit irrigation impacts on greenhouse gas emissions under drip-fertigated maize in the Great Plains of Colorado. J. Environ. Qual..

[26] Davis, J. G., & Westfall, D. G. Fertilizing Corn, Fact Sheet No. 0.538. Colorado State University Extension. 0, 3–7 (2014).

[27] Doane TA, Horwáth WR (2003). Spectrophotometric determination of nitrate with a single reagent. Anal. Lett..

[28] Hutchinson GL, Mosier AR (1981). Improved soil cover method for field measurement of nitrous oxide fluxes. Soil Sci. Soc. Am. J..

[29] Nichols KL (2016). Nitrous oxide and methane fluxes from cattle excrement on C3 pasture and C4-dominated shortgrass steppe. Agric. Ecosyst. Environ..

[30] Trout TJ, DeJonge KC (2017). Water productivity of maize in the US high plains. Irrig. Sci..

[31] Parkin, T. B., & Venterea, R. T. Chapter 3. Chamber-based trace gas flux measurements. In *USDA-ARS GRACEnet Project Protocols* 1–39 (2010).

[32] Bates D, Mächler M, Bolker B, Walker S (2015). Fitting linear mixed-effects models using lme4. J. Stat. Softw..

[33] R Core Team. *R: A Language and Environment for Statistical Computing* (R Foundation for Statistical Computing, 2020).

[34] Goyal, S. S., & Huffaker, R. C. Nitrogen toxicity in plants. In *Nitrogen in Crop Production* (ed. Hauck, R. D.) 97–118 (1984).

[35] Moser SB, Feil B, Jampatong S, Stamp P (2006). Effects of pre-anthesis drought, nitrogen fertilizer rate, and variety on grain yield, yield components, and harvest index of tropical maize. Agric. Water Manag..

[36] Wang Y, Janz B, Engedal T, Neergaard AD (2017). Effect of irrigation regimes and nitrogen rates on water use efficiency and nitrogen uptake in maize. Agric. Water Manag..

[37] Comas LH, Becker SR, Cruz VMV, Byrne PF, Dierig DA (2013). Root traits contributing to plant productivity under drought. Front. Plant. Sci..

[38] Flynn N, Comas LH, Stewart CE, Fonte SJ (2021). Deficit irrigation drives maize root distribution and soil microbial communities with implications for soil carbon dynamics. Soil Sci. Soc. Am. J..

[39] Gardner JB, Drinkwater LE (2009). The fate of nitrogen in grain cropping systems: A meta-analysis of 15N field experiments. Ecol. Appl..

[40] Yan M, Pan G, Lavallee JM, Conant RT (2020). Rethinking sources of nitrogen to cereal crops. Glob. Change Biol..

[41] Nasielski J, Earl H, Deen B (2019). Luxury vegetative nitrogen uptake in maize buffers grain yield under post-silking water and nitrogen stress: A mechanistic understanding. Front. Plant Sci..

[42] Song Y (2019). Nitrogen increases drought tolerance in maize seedlings. Funct. Plant Biol..

[43] Schirrmacher V (2021). Less can be more: The hormesis theory of stress adaptation in the global biosphere and its implications. Biomedicines..

[44] Chen Y (2015). Effects of nitrogen application on post-silking root senescence and yield of maize. Agronomy J..

[45] Liu Z (2017). Effects of integrated agronomic practices management on root growth and development of summer maize. Eur. J. Agron..

[46] Al-Kaisi M, Yin X (2003). Effects of nitrogen rate, irrigation rate, and plant population on corn yield and water use efficiency. J. Agron..

[47] Payero JO, Melvin SR, Irmak S, Tarkalson D (2006). Yield response of corn to deficit irrigation in a semiarid climate. Agric. Water Manag..

[48] Rudnick, D. *et al*. Deficit irrigation management of corn in the high plains: A review. In *29th Annual Central Plains Irrigation Conference* 66–84 (2017).

[49] Gheysari M, Majid S, Homaee M (2009). Nitrate leaching in a silage maize field under different irrigation and nitrogen fertilizer rates. Agric. Water Manag..

[50] Teixeira EI (2014). The impact of water and nitrogen limitation on maize biomass and resource-use efficiencies for radiation, water and nitrogen. Field Crops Res..

[51] Gärdenäs AI, Hopmans JW, Hanson BR, Šimůnek J (2005). Two-dimensional modeling of nitrate leaching for various fertigation scenarios under micro-irrigation. Agric. Water Manag..

[52] Hanson BR, Šimůnek J, Hopmans JW (2006). Evaluation of urea-ammonium-nitrate fertigation with drip irrigation using numerical modeling. Agric. Water Manag..

[53] Goulding K (2000). Nitrate leaching from arable and horticultural land. Soil Use Manag..

[54] Endelman FJ, Keeney DR, Gilmour JT, Saffigna PG (1974). Nitrate and chloride movement in the plainfield loamy sand under intensive irrigation. J. Environ. Qual..

[55] Klocke NL (1999). Nitrate leaching in irrigated corn and soybean in a semi-arid climate. Trans. ASABE..

[56] Baggs EM, Rees RM, Smith KA, Vinten AJ (2000). Nitrous oxide emission from soils after incorporating crop residues. Soil Use Manag..

[57] Abalos D, Sanchez-Martin L, Garcia-Torres L, van Groenigen JW, Vallejo A (2014). Management of irrigation frequency and nitrogen fertilization to mitigate GHG and NO emissions from drip-fertigated crops. Sci. Total Environ..

[58] Signor D, Cerri CEP (2013). Nitrous oxide emission in agricultural soils: A review. Pesqui. Agropecu. Trop..

[59] Bremner JM (1997). Sources of nitrous oxide in soils. Nutr. Cycl. Agroecosyst..

[60] Snyder CS, Bruulsema TW, Jensen TL, Fixen PE (2009). Review of greenhouse gas emissions from crop production systems and fertilizer management effects. Agric. Ecosyst. Environ..

[61] Cameron KC, Di HJ, Moir JL (2013). Nitrogen losses from the soil/plant system: A review. Ann. Bot..

[62] Adviento-Borbe MAA, Haddix ML, Binder DL, Walters DT, Dobermann A (2007). Soil greenhouse gas fluxes and global warming potential in four high-yielding maize systems. Glob. Change Biol..

[63] Guardia G (2017). Effect of inhibitors and fertigation strategies on GHG emissions, NO fluxes and yield in irrigated maize. Field Crops Res..

[64] Halvorson AD, Del Grosso SJ, Stewart CE (2016). Manure and inorganic nitrogen affect trace gas emissions under semi-arid irrigated corn. J. Environ. Qual..

[65] Sanz-Cobena A (2017). Strategies for greenhouse gas emissions mitigation in Mediterranean agriculture: A review. Agric. Ecoyst. Environ..

