# Supplementary material for: High N availability decreases N uptake and yield under limited water availability in maize
Source: Sci Rep. 2023 Aug 31;13:14269. doi: 10.1038/s41598-023-40459-0 (PMC10471730; doi:10.1038/s41598-023-40459-0)
Supplement: Supplementary file 1 — Supplementary Figures. [file 41598_2023_40459_MOESM1_ESM.docx]

**Supplementary Information**


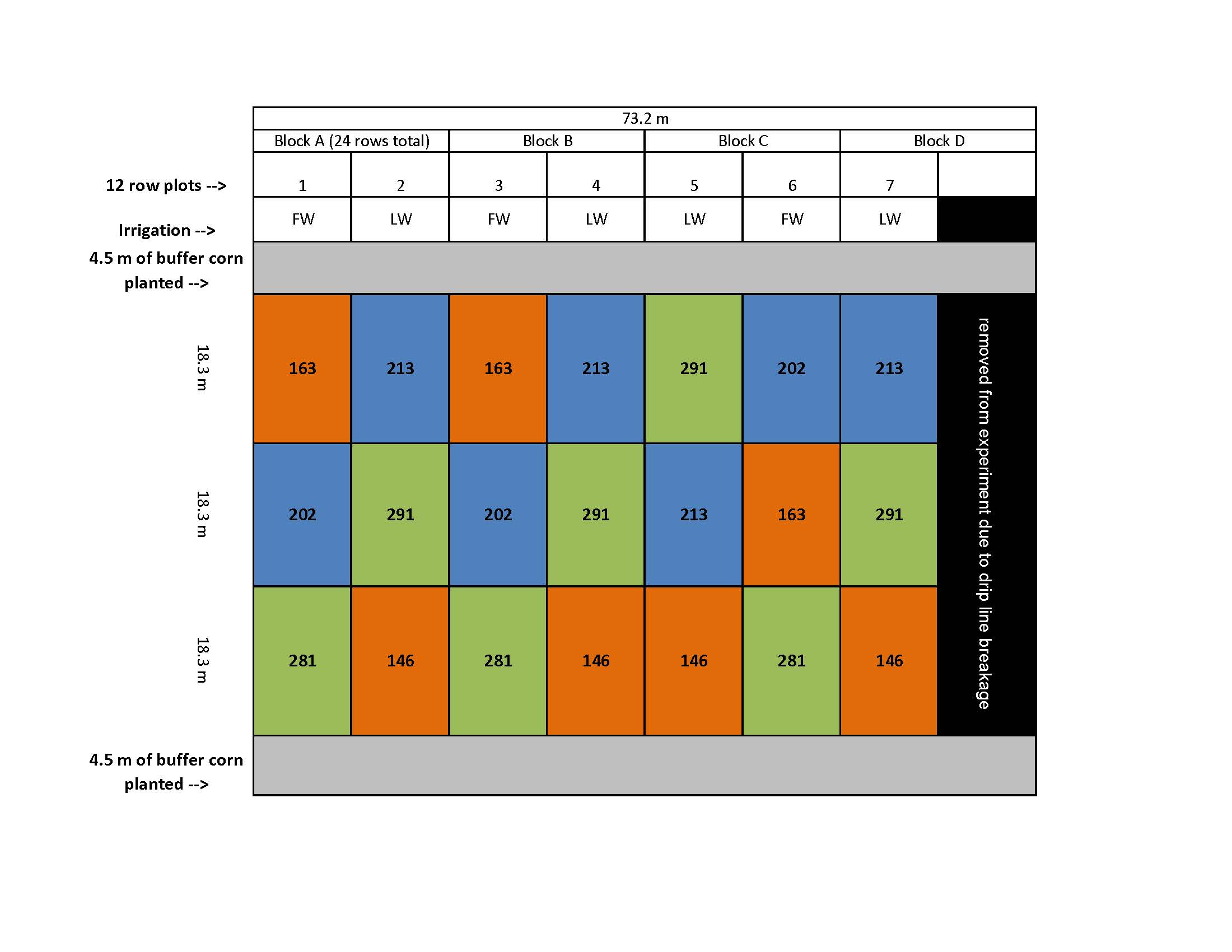


**Figure S1.** Description of the randomized split-plot experimental layout used in this study. Four replicated blocks had full and limited water treatments randomly assigned to 12 row planted sections of each block. One 12-row section was omitted because of breakage in the drip irrigation line, leaving 3 replicates of the full water treatment and four replicates of the limited water treatment. Three nitrogen (N) levels were randomized within each water section, but the amount of N fertilizer added at the beginning of the season was determined to account for the estimated amount of N that would also be added from the irrigation over the course of the season. The values in each plot indicate the amount of N applied in total (kg ha^-1^) over the season from fertilizer and the irrigation water.


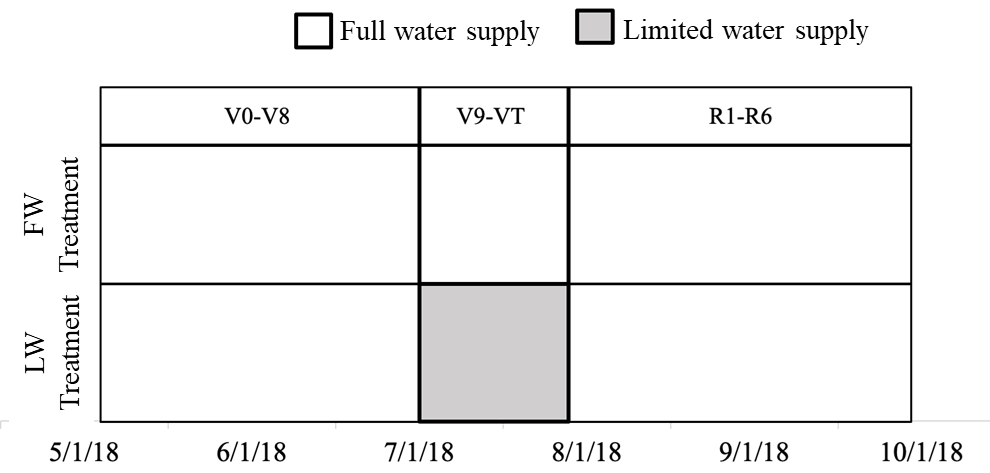


**Figure S2.** Description of the water treatments used in this study. Less than full irrigation (75% of full ET) was supplied for the limited water treatment from July 3, 2018, to July 31, 2018, during the late vegetative (V9-VT) growth phase with full irrigation supplied to meet the crop’s water requirement (100% of full ET) before and after this period. For the full water treatment, the crop’s full water requirement was supplied during the entire growing season.
